# Retrospective analysis of the endometrial preparation protocols for frozen-thawed embryo transfer cycles in women with endometriosis

**DOI:** 10.1186/s12958-023-01132-3

**Published:** 2023-09-05

**Authors:** Jingdi Yang, Yangxing Wen, Danping Li, Xuerong Hou, Bo Peng, Zengyan Wang

**Affiliations:** 1 Reproductive Center, Guangdong Key Laboratory of Reproductive Medicine, The First Affiliated Hospital of Sun Yat-sen University, No. 58 Zhongshan Er Road, 510080 Guangzhou, China; 2 Department of Rehabilitation Medicine, The First Affiliated Hospital of Sun Yat-sen University, Guangzhou, China

**Keywords:** Endometrial preparation protocols, Endometriosis, GnRHa, Pregnancy outcomes

## Abstract

**Background:**

There was inconsistency in optimal endometrial preparation protocol for frozen-thawed embryo transfer (FET) in patients with endometriosis. We conducted this study to investigate the effect of different endometrial preparation protocols on the pregnancy outcomes in patients with endometriosis undergoing FET cycles, and determine the optimal number of GnRHa injections in GnRHa-HRT protocols.

**Method(s):**

This was a retrospective cohort analysis of women with endometriosis who underwent FET cycles at a single university-based center. This study retrospectively analyzed 2048 FET cycles in our center from 2011 to 2020. According to the endometrial preparation protocols, patients were divided into 4 groups: gonadotropin releasing hormone agonist-hormone replacement therapy(GnRHa-HRT), hormone replacement therapy(HRT), ovulation induction(OI), and natural cycle(NC). In the GnRHa-HRT group, patients were further divided into 3 groups: one injection of GnRHa, two injections of GnRHa, and three or more injections of GnRHa. The primary outcome was the clinical pregnancy rate. Propensity score matching was used to adjust for potential non-similarities among the groups. Multivariate logistic regression analysis was performed to figure out the risk factors for pregnancy outcomes.

**Result(s):**

There were no statistical differences in pregnancy outcomes among the four endometrial preparation protocols in FET cycles with endometriosis patients, the results retained after propensity score matching(PSM). And in endometriosis patients complicated with adenomyosis, the results remained similar. In patients with GnRHa-HRT protocol, there were no differences in clinical pregnancy rate and live birth rate with different numbers of GnRHa injections, the early miscarriage rate were 18% in the two injections of GnRHa group and 6.5% in the one injection of GnRHa group(P = 0.017). Multifactorial logistic regression analysis showed that two injections of GnRHa before FET was associated with increased early miscarriage rate compared with one injection of GnRHa[adjusted OR (95% CI): 3.116(1.079–8.998),p = 0.036].

**Conclusion(s):**

The four kinds of endometrial preparation protocols for FET, GnRHa-HRT, HRT, OI and NC had similar pregnancy outcomes in patients with endometriosis. In endometriosis patients complicated with adenomyosis, the results remained similar. In patients with endometriosis undergoing GnRHa-HRT protocol for FET, more injections of GnRHa had no more advantages in pregnancy outcomes, on the contrary, it might increase the early miscarriage rate.

## Background

Endometriosis is the presence of the endometrium outside the uterine cavity, with persistent growth and infiltration of ectopic tissues, which then causes inflammation, pain, and infertility [1]. Endometriosis is a common condition in women of reproductive age, with 5–10% of women of reproductive age suffering from endometriosis and 40–50% of women with endometriosis also suffering from infertility [2]. The European Society of Human Reproduction and Embryology(ESHRE) guidelines for endometriosis recommended that assisted reproductive technology(ART) could be applied to endometriosis patients with infertility [3, 4].

GnRH-a blocks pituitary GnRH release to hinder ovary hormone secretion, while preventing premature luteinization of follicles and enhancing follicular growth synchronization [5]. Some studies suggested GnRH-a might improve the pelvic environment and yield high-quality eggs and embryos in patients with endometriosis [5, 6]. However, the GnRHa-HRT protocol has disadvantages such as high cost [7], risk of hypo-estrogenic side effects [8], ovarian cyst formation [7, 9, 10], and a time-consuming preparation process [11]. The effectiveness of GnRHa in endometrial preparation for FET cycles was controversial, with no definitive conclusions on the preferred protocol for patients with endometriosis. A meta-analysis showed that downregulation was effective only for stage III or IV endometriosis patients, but not for mild endometriosis patients [12]. However, a retrospective analysis found no significant differences in pregnancy outcomes among NC, HRT, and GnRHa-HRT cycle protocols for patients with endometriosis undergoing FET [13].

Patients with endometriosis may also be diagnosed with adenomyosis, adenomyosis is an independent risk factor for low fertility in endometriosis patients [14]. There was no consensus on the use of GnRHa prior to FET for patients with adenomyosis, different studies have reached opposite conclusions [15–17].

The number of GnRHa injections before FET in GnRHa-HRT protocol varies among hospitals and clinicians [12]. The effectiveness of long-term GnRHa treatment before IVF/ICSI in endometriosis patients remains uncertain [18, 19]. A randomized controlled study found no improvement in pregnancy outcomes with the ultralong protocol in patients with endometriosis, meanwhile found a longer duration of ovarian stimulation, higher consumption of gonadotrophins, and lower ovarian estradiol production [20]. Additionally, another study found that a shortened number of GnRHa applications for IVF was found to be as effective as an extra-long protocol [21]. The ESHRE guideline for endometriosis did not recommend extended use of GnRH agonist therapy to improve live birth rates due to uncertain benefit [4].

There was inconsistency in endometrial preparation protocols for FET in patients with endometriosis and adenomyosis. Can GnRHa downregulation improve the clinical outcomes of the FET cycle in patients with endometriosis and adenomyosis? And what about increasing the number of GnRHa injections? To answer these questions, we retrospectively analyzed 2048 FET cycles in endometriosis patients from 2011 to 2020, and investigated the effect of different endometrial preparation protocols on pregnancy outcomes. Pregnancy outcomes of endometriosis patients complicated with adenomyosis undergoing FET were also explored. Then, we further explored the effect of the numbers of GnRHa injections used before FET on pregnancy outcomes in GnRHa-HRT endometrial preparation protocol.

## Materials and methods

### Study design

This was a retrospective cohort analysis of women with endometriosis who underwent FET cycles. Women with endometriosis were treated with FET cycles at the Reproductive Medicine center, The First Affiliated Hospital of Sun Yat-sen University. The institutional ethics committee (The First Affiliated Hospital of Sun Yat-sen University) approved the study, and informed consent was waived due to the retrospective nature of this study.

### Patient inclusion

This study retrospectively analyzed 2048 FET cycles of patients with endometriosis in our center from 2011 to 2020. Patients included in the analysis were diagnosed with endometriosis by laparoscopic or laparotomy, or in some cases diagnosed by transvaginal/rectal ultrasound suggestive of ovarian endometriotic cysts. The inclusion criteria were: (1) patients with a diagnosis of endometriosis who underwent FET at our center, (2) female age at FET: <40 years. The exclusion criteria were: (1) abnormal uterine cavity, (2) recurrent miscarriages, and (3) repeated implantation failure (Supplemental Fig. 1).

To compare the efficacy of different endometrial preparation protocols during FET cycles for endometriosis patients, four groups were analyzed: GnRHa-HRT, HRT, OI, and NC. Baseline characteristics as well as pregnancy outcomes were compared. Propensity score matching(PSM) was used to adjust for potential non-similarities among the groups and determine if the GnRHa-HRT protocol was superior in terms of pregnancy outcomes. PSM matched baseline characteristics and other variables using logistic regression model. For endometriosis patients complicated with adenomyosis were also compared. In the GnRHa-HRT protocol, patients were further grouped based on the number of GnRHa used before FET, and the baseline characteristics and pregnancy outcomes were compared.

### Endometrial preparation protocols

The endometrial preparation protocols in this study include GnRHa-HRT, HRT, OI, and NC. Clinicians chose a protocol based on the patient’s condition and their own experiences, but there is no strict and unified standard in clinical practice.

### FET with GnRHa-HRT and HRT protocols

Usually the GnRHa-HRT protocol was used if the patient was diagnosed with ovarian endometriotic cyst or if there was a combination of adenomyosis. GnRHa Triptorelin Acetate Injection(Decapeptyl; Ferring GmbH, Germany) was administered on the second day of the menstrual cycle(D2) for down regulation, the dosage of GnRHa ranges from 0.8 to 3.75 mg, with 1 mg, 1.3 mg, 1.8 mg and 3.75 mg being the most commonly used, depending on the patient’s condition and the practice of different physicians. And the decision to administer the next GnRHa injection was made after an interval of 28 days(rarely 14 days), a second GnRHa injection is given if the the size of ovarian endometriosis cyst or adenomyosis lesion has not decreased. Oral oestradiol(E2) valerate (Progynova; Bayer Schering Pharma AG, Berlin, Germany) was administered for 10–18 days from day 2–3 of menstruation, transvaginal ultrasonography was performed, if there was no sign of a dominant follicle or ovulation and the endometrial thickness reached at least 7 mm, conversion of the endometrium was determined based on the endometrial condition and hormone levels, then FET was planned. Luteal support was started on the same day and embryo transfer was performed on day 4 or 6 depending on embryo development, E2 could continue to be used. If pregnancy was confirmed, E2 and progesterone(P, Dydrogesterone, Abbott, USA) supplement would be continued until about 10 weeks of pregnancy. The FET protocol with HRT was similar to that with GnRHa-HRT, except that GnRHa was not used, this protocol was commonly used for women with irregular menstruation or a history of anovulation.

### FET with NC protocol

NC protocol was a favorable option for women with regular menstruation. Usually modified NC protocol was used. Transvaginal ultrasound and basal hormone levels test were performed from day 2 to 3 of the menstrual cycle. On day 8 to 10 of the menstrual cycle, ultrasound was performed to monitor follicle size, endometrial thickness and morphology. At the same time, the patient would undergo urine luteinizing hormone(LH) test strip by their own. If a strong positive was found, blood E2, P and LH were measured, and confirmed the LH peak. After the LH peak, the ultrasound monitoring still continued, the ovulation day was set as Day one (D1). And luteal support was started on the day of ovulation, and embryo transfer was performed on the third or fifth day of initiation of P administration(D3 or D5) for cleavage embryos or blastocysts. In order to prevent luteal insufficiency, we routinely used luteal support, Dydrogesterone 10 mg po BID, for 17 days to the pregnancy test date, if pregnant, the drug was continued, if not pregnant, the drug was stopped.

### FET with OI protocol

OI protocol might be considered when patients were combined with polycystic ovary syndrome(PCOS) or other reasons caused ovulatory dysfunction. Ultrasound monitoring was started on the third to fifth day of menstruation, if the endometrial thickness ≤ 5 mm and there were no dominant follicles in both ovaries, OI protocol was performed. Letrozole (Novartis, Sweden) 2.5 mg BID for 5 days or clomiphene(Kangtai, China) 50/100 mg QD for 5 days was given, follicles were monitored under ultrasound after 5 days, human menopausal Gonadotropin(HMG) 37.5-75IU was given, when the dominant follicle was ≥ 18 mm, human chorionic gonadotropin(HCG) was injected intramuscularly, ovulation was confirmed by ultrasound after 36 h, after confirming ovulation, luteal support was given, embryo transfer was performed on the third or fifth day of initiation of P administration. The luteal support protocol was similar with the above NC protocol.

### Outcomes

The primary outcome in this study was the clinical pregnancy rate. Secondary outcomes included live birth rate, early miscarriage rate. The clinical pregnancy was defined as a gestational sac detected by ultrasound after 4 weeks of embryo transfer. Live birth was defined as delivery with at least one live fetus. Early miscarriage was defined as miscarriage occurring before 12 weeks of gestation.

### Statistical analyses

Statistical analysis was performed using SPSS 23. 0 software. The continuous variables were expressed as ( mean ± standard deviation), and the Kolmogorov-Smirnov test was used for normality analysis, and the data conforming to normal distribution were compared using one-way analysis of variance, and the non-normally distributed data were compared using Kruskal-Wallis test. The categorical data were expressed as frequency and percentage which were compared among groups using the chi-square test or Fisher’s exact test. To exclude confounding factors logistic regression analysis was performed, the covariates included in the analysis were: baseline characteristics and other variables that may affect pregnancy outcomes. All covariates were first analyzed separately by univariate logistic regression, and covariates with P value < 0.2 were included in the multivariate logistic regression analysis. Some variables of our interest and clinically considered important such as: endometrial preparation protocols, maternal age at FET, infertility diagnosis, BMI, endometriosis American Society for Reproductive Medicine(ASRM) stage [22], and the proportion of endometriosis complicated with adenomyosis were always included in the multifactorial logistic regression analysis. Finally, 1:1 matching was performed using PSM, and propensity scores were calculated based on baseline characteristics and other variables that may affect pregnancy outcomes using logistic regression models. P values less than 0.05 were considered to be statistically significant.

## Results

### Baseline and cycle characteristics

According to the inclusion and exclusion criteria, 2048 FET cycles of patients with endometriosis in our center from 2011 to 2020 were analyzed, 451, 696, 42, and 859 cycles were included in the GnRHa-HRT, HRT, OI, and NC endometrial preparation protocols, respectively. The details of the patients’ baseline characteristics were shown in Table 1. There were statistically significant differences in the duration of infertility, infertility diagnosis, infertility etiology(tubal factors, other factors), endometriosis ASRM stage, and the proportion of endometriosis patients complicated with adenomyosis (P < 0.05).

**Table 1:** General characteristics and clinical outcomes of patients with different endometrial preparation protocols

Variables			1:GnRH-a-HRT	2:HRT	3:OI	4:NC	P value
**No. of cases**			451	696	42	859	-
**Maternal age at FET, years**			32.29 ± 3.49	32.11 ± 3.53	32.71 ± 3.49	32.29 ± 3.40	0.551
**Duration of infertility, years**			3.50 ± 2.34	4.02 ± 2.67	3.74 ± 2.36	4.12 ± 2.68	0.001^a^
**Body mass index, kg/m2**			20.75 ± 2.45	20.80 ± 2.48	21.25 ± 2.83	20.69 ± 2.42	0.607
**Infertility diagnosis**	**Primary infertility, n (%)**		61.2% (276/451)	54.7% (381/696)	40.5% (17/42)	59.4% (510/859)	0.001^a^
	**Secondary infertility, n (%)**		36.1%(163/451)	43.5%(303/696)	54.8%(23/42)	40%(344/859)	
	**Infertility-free** ^**b**^		2.7%(12/451)	1.7%(12/696)	4.8%(2/42)	0.6%(5/859)	
**Infertility etiology, n (%)**	**Tubal factor**		51.2%(231/451)	56.8%(395/696)	61.9%(26/42)	63.7%(547/859)	0.000^a^
	**Male factor**		49.7%(224/451)	47.6%(331/696)	57.1%(24/42)	47.4%(407/859)	0.556
	**Other**		33.9%(153/451)	35.5%(247/696)	19%(8/42)	24.9%(214/859)	0.000^a^
**endometriosis ASRM stage** ^**c**^	**ASRMI-II**		14.6%(66/451)	45.7%(318/696)	50%(21/42)	51.2%(440/859)	0.000^a^
	**ASRMIII-IV**		85.4%(385/451)	54.3%(378/696)	50%(21/42)	48.8%(419/859)	
**endometriosis complicated with adenomyosis**			20.4%(92/451)	3.6%(25/696)	14.3%(6/42)	3.3%(28/859)	0.000^a^
**Ovarian stimulation protocols, n (%)**	**Long GnRH-a**		55.2%(249/451)	74.1%(516/696)	69%(29/42)	79.6%(684/859)	0.000^a^
	**GnRH-a ultra-long**		12.4%(56/451)	4%(28/696)	0%(0/42)	2.1%(18/859)	
	**GnRH antagonist**		24.8%(112/451)	15.1%(105/696)	26.2%(11/42)	12.2%(105/859)	
	**Other protocols**		7.5%(34/451)	6.8%(47/696)	4.8%(2/42)	6.1%(52/859)	
**Gonadotropin dose, IU**			2621 ± 945	2325 ± 894	2748 ± 1038	2300 ± 848	0.000^a^
**No. of oocytes retrieved**			12.15 ± 7.53	15.09 ± 8.9	14.5 ± 9.35	15.29 ± 8.43	0.000^a^
**Blastocyst formation rate**			0.60 ± 0.28	0.56 ± 0.28	0.59 ± 0.28	0.56 ± 0.29	0.313
**Type of embryo transferred, n (%)**		**Cleavage embryo**	45.0%(203/451)	42.1%(293/696)	35.7%(15/42)	45.5%(391/859)	0.365
		**Blastocyst**	55.0%(248/451)	57.9%(403/696)	64.3%(27/42)	54.5%(468/859)	
**No. of embryos transferred**			1.58 ± 0.57	1.71 ± 0.65	1.48 ± 0.51	1.78 ± 0.68	0.000^a^
**No. of good quality embryos transferred**			1.15 ± 0.69	1.13 ± 0.78	0.93 ± 0.75	1.12 ± 0.78	0.287
**Rate of good quality embryos transferred**			83.8%(378/451)	78.3%(545/696)	69%(29/42)	78.9%(678/859)	0.033^a^
**Clinical Pregnancy**			53.2%(240/451)	49.7%(346/696)	54.8%(23/42)	46.8%(402/859)	0.141
**Live Birth**			43.5%(196/451)	39.8%(277/696)	45.2%(19/42)	39.0%(335/859)	0.397
**Early Miscarriage**			15.4%(37/240)	18.2%(63/346)	17.4%(4/23)	15.4%(62/402)	0.732

Note: ^a^P<0.05

^b^Infertility-free: Patients who did not meet the diagnostic criteria for infertility, mainly included patients treated with preimplantation genetic testing(PGT)

^c^endometriosis ASRM stage: The American Society of Reproductive Medicine distinguishes four stages of endometriosis, where stage I and II are fairly mild types, and stages III and IV are advanced disease

Among the cycle characteristics, there were statistical differences in ovarian stimulation protocols, gonadotropin (Gn) dose, number of oocytes retrieved, number of embryos transferred, and rate of high quality embryos transferred (P < 0.05) (Table 1).

### Pregnancy outcomes

Pregnancy outcomes were shown in Table 1, with no significant differences in clinical pregnancy rate, live birth rate, or early miscarriage rate among the 4 endometrial preparation protocols. Baseline and cycle characteristics were analyzed by univariate and multivariate logistic regression with pregnancy outcomes as the dependent variables. After adjusting for possible confounders, no correlation was shown among different endometrial preparation protocols and pregnancy outcomes(Table 2).

**Table 2:** Adjusted odds ratios of clinical outcomes

Variable	GnRHa-HRT vs. NC Adjusted OR (95% CI)	GnRHa-HRT vs. HRT Adjusted OR (95% CI)	HRT vs. NC Adjusted OR (95% CI)	OI vs. NC Adjusted OR (95% CI)
**Clinical Pregnancy** ^**a**^	1.166(0.904–1.503)	1.072(0.828–1.387)	1.088(0.885–1.336)	1.347(0.711–2.551)
**Live Birth** ^**b**^	1.16(0.897-1.5)	1.157(0.892–1.502)	1.002(0.813–1.236)	1.272(0.671–2.41)
**Early Miscarriage** ^**c**^	0.811(0.499–1.318)	0.735(0.466–1.16)	1.23(0.833–1.816)	1.119(0.361–3.464)

Note: Adjusted odds ratios (AORs) and 95% CIs are based on the multiple logistic regression model. No statistical difference for all results. P > 0.05 for all comparisons

^a^Adjusted for Maternal age at FET, infertility diagnosis, BMI, endometriosis ASRM stage, endometriosis combined with adenomyosis, Infertility etiology (tubal factor, other), duration of infertility, No. of high quality embryos transferred, rate of high quality embryos transferred, Ovarian stimulation protocols, No. of oocytes retrieved, blastocyst formation rate

^b^Adjusted for Maternal age at FET, infertility diagnosis, BMI, endometriosis ASRM stage, endometriosis combined with adenomyosis, Infertility etiology (tubal factor), duration of infertility, No. of high quality embryos transferred, rate of high quality embryos transferred, Ovarian stimulation protocols, No. of oocytes retrieved, blastocyst formation rate

^c^Adjusted for Maternal age at FET, infertility diagnosis, BMI, endometriosis ASRM stage, endometriosis combined with adenomyosis, duration of infertility, blastocyst formation rate

### PSM

After PSM, 312 cycles with GnRHa-HRT protocol and 312 cycles with NC protocol were compared, there were no statistical differences in the baseline characteristics and other variables that may affect pregnancy outcomes between the two groups, and further analysis also showed no statistical differences in pregnancy outcomes between the two groups (Table 3). Three hundred and thirty-two cycles with GnRHa-HRT protocol and 332 cycles with HRT protocol were compared, there were no statistical differences in most of the baseline characteristics between the two groups after matching, and no statistical differences in pregnancy outcomes between the two groups(Table 3). There were 42 cases in the OI group which did not perform PSM due to the small number of cases.

**Table 3:** General characteristics and pregnancy outcomes of patients with different endometrial preparation protocols after PSM

			NC and GnRHa-HRT after PSM			HRT and GnRHa-HRT after PSM		
			NC	GnRHa-HRT	P value	HRT	GnRHa-HRT	P value
**No. Of cases**			312	312	-	332	332	-
**Maternal age at FET, years**			32.27 ± 3.46	32.18 ± 3.56	0.959	32.12 ± 3.47	32.3 ± 3.52	0.479
**Duration of infertility, years**			3.68 ± 2.44	3.8 ± 2.5	0.562	3.61 ± 2.56	3.68 ± 2.47	0.542
**Body mass index, kg/m2**			20.52 ± 2.36	20.6 ± 2.47	0.52	20.73 ± 2.27	20.66 ± 2.38	0.707
**Infertility diagnosis**	**Primary infertility, n (%)**		60.6%(189/312)	63.5%(198/312)	0.73	59.3%(197/332)	59.3%(197/332)	0.969
	**Secondary infertility, n (%)**		38.1%(119/312)	35.6%(111/312)		38%(126/332)	38.3%(127/332)	
	**Infertility-free**		1.3%(4/312)	1%(3/312)		2.7%(9/332)	2.4%(8/332)	
**Infertility etiology, n (%)**	**Tubal factor**		58%(181/312)	54.2%(169/312)	0.333	55.4%(184/332)	50.9%(169/332)	0.243
	**Male factor**		47.8%(149/312)	47.4%(148/312)	0.936	52.4%(174/332)	50.6%(168/332)	0.641
	**Other**		27.9%(87/312)	30.8%(96/312)	0.429	32.8%(109/332)	34.3%(114/332)	0.681
**endometriosis ASRM stage**	**ASRMI-II**		22.1%(69/312)	21.2%(66/312)	0.771	18.7%(62/332)	19.6%(65/332)	0.767
	**ASRMIII-IV**		77.9%(243/312)	78.8%(246/312)		81.3%(270/332)	80.4%(267/332)	
**endometriosis complicated with adenomyosis**			6.7%(21/312)	6.1%(19/312)	0.744	6.9%(23/332)	6%(20/332)	0.636
**Ovarian stimulation protocols, n (%)**	**Long GnRH-a**		66%(206/312)	66.3%(207/312)	0.986	64.5%(214/332)	64.2%(213/332)	0.951
	**GnRH-a** **ultra-long**		5.1%(16/312)	4.8%(15/312)		6.9%(23/332)	6.3%(21/332)	
	**GnRH antagonist**		21.2%(66/312)	20.5%(64/312)		19.9%(66/332)	21.4%(71/332)	
	**Other protocols**		7.7%(24/312)	8.3%(26/312)		8.7%(29/332)	8.1%(27/332)	
**Gonadotropin dose, IU**			2338.82 ± 878.17	2532.28 ± 966.03	0.009^a^	2471.42 ± 936.87	2533.49 ± 979.57	0.433
**No. of oocytes retrieved**			12.99 ± 7.84	13.37 ± 7.84	0.358	13.08 ± 8.15	12.6 ± 7.76	0.615
**Blastocyst formation rate**			0.61 ± 0.29	0.59 ± 0.26	0.389	0.60 ± 0.27	0.60 ± 0.28	0.936
**Type of embryo transferred, n (%)**		**Cleavage embryo**	44.9%(140/312)	45.5%(142/312)	0.872	47.9%(159/332)	47.6%(158/332)	0.938
		**Blastocyst**	55.1%(172/312)	54.5%(170/312)		52.1%(173/332)	52.4%(174/332)	
**No. of embryos transferred**			1.69 ± 0.66	1.59 ± 0.56	0.036^a^	1.71 ± 0.62	1.60 ± 0.55	0.018^a^
**No. of good quality embryos transferred**			1.11 ± 0.69	1.14 ± 0.71	0.573	1.18 ± 0.69	1.17 ± 0.71	0.814
**Rate of good quality embryos transferred**			81.7%(255/312)	82.4%(257/312)	0.835	84.3%(280/332)	83.1%(276/332)	0.674
**Clinical Pregnancy**			48.1%(150/312)	53.5%(167/312)	0.173	52.1%(173/332)	50.9%(169/332)	0.756
**Live Birth**			41.7%(130/312)	43.6%(136/312)	0.627	42.2%(140/332)	43.1%(143/332)	0.814
**Early Miscarriage**			12.7%(19/150)	15%(25/167)	0.554	17.9%(31/173)	11.8%(20/169)	0.114

Note: ^a^P<0.05

### Endometriosis complicated with adenomyosis

There were 151 cycles (17.6%) complicated with adenomyosis, and adenomyosis was classified according to severity as focal or diffuse. There were no significant differences in pregnancy outcomes among the different endometrial preparation protocols(Supplemental Table 1). After univariate and multivariate regression analysis, the results were still retained(Supplemental Table 2).

### Effect of the number of GnRHa injections before FET on pregnancy outcomes

The GnRHa-HRT endometrial preparation protocol consisted of 451 cycles, which could be divided into 3 groups according to the number of GnRHa-HRT jnjections before FET. For the baseline characteristics, the proportion of endometriosis complicated with adenomyosis and endometriosis ASRM stage were statistically different among the three groups(P < 0.05). About the cycle characteristics, ovarian stimulation protocols, Gn dosage, number of oocytes retrieved, type of embryo transferred and number of high quality embryos transferred were statistically different among the three groups (P < 0.05) (Supplemental Table 3).

Pregnancy outcomes were analyzed, and there were no statistical differences in clinical pregnancy rate and live birth rate among the three groups, the early miscarriage rate were 18% in the two GnRHa injections group and 6.5% in one GnRHa injection group (P = 0.017) (Supplemental Table 3). Subsequent univariate and multifactorial logistic analyses showed that there were no correlations between the number of GnRHa injections and clinical pregnancy rate as well as live birth rate, two GnRHa injections before FET was associated with increased early miscarriage rate compared with one GnRHa injection[adjusted OR (95% CI): 3.116(1.079–8.998),p = 0.036] (Table 4).

**Table 4:** **A**djusted odds ratios of pregnancy outcomes in endometriosis patients with GnRHa-HRT endometrial preparation protocol

Variable	Two injections of GnRHa vs. One injection of GnRHa	Three injections of GnRHa vs. One injection of GnRHa	Two injections of GnRHa vs. Three injection of GnRHa
	Adjusted OR (95% CI)	Adjusted OR (95% CI)	Adjusted OR (95% CI)
**Clinical Pregnancy** ^**a**^	0.867(0.533–1.412)	0.887(0.431–1.828)	0.977(0.444–2.154)
**Live Birth** ^**b**^	0.641(0.391–1.052)	0.642(0.305–1.35)	0.999(0.442–2.261)
**Early Miscarriage** ^**c**^	3.116(1.079–8.998)^d^	2.472(0.644–9.488)	1.26(0.298–5.34)

Note: Adjusted odds ratios (AORs) and 95% CIs are based on the multiple logistic regression model. No statistical difference for all results

^a^Adjusted for Maternal age at FET, endometrial preparation protocols, infertility diagnosis, BMI, endometriosis ASRM stage, endometriosis combined with adenomyosis, rate of high quality embryos transferred, Ovarian stimulation protocols, No. of oocytes retrieved, blastocyst formation rate

^b^Adjusted for Maternal age at FET, endometrial preparation protocols, infertility diagnosis, BMI, endometriosis ASRM stage, endometriosis combined with adenomyosis, infertility etiology (tubal factor, other factors), No. of oocytes retrieved, blastocyst formation rate

^c^Adjusted for Maternal age at FET, endometrial preparation protocols, infertility diagnosis, BMI, endometriosis ASRM stage, endometriosis combined with adenomyosis, infertility etiology (tubal factor, male factor, other factors), No. of high quality embryos transferred, Gonadotropin dose

^d^P<0.05

## Discussion

FET is commonly used in ART due to various reasons including unsuitability of endometrial for fresh cycle transfer, high risk of ovarian hyperstimulation syndrome in fresh cycles, and promotion of cumulative live birth rate [23]. The rate of FET performed in patients with endometrosis was about 41% and the rate of fresh transplantation was about 59% [24]. However, the best endometrial preparation protocol for FET remained uncertain. Our study compared four endometrial preparation protocols in patients with endometriosis and found no significant difference in pregnancy outcomes. After PSM analysis the results remained similar. For endometriosis patients complicated with adenomyosis, there were also no significant differences in pregnancy outcomes among the four protocols. In patients undergoing GnRHa-HRT protocol, there were no significant differences in clinical pregnancy and live birth rates among those with different numbers of GnRHa used, but two injections of GnRHa increased early miscarriage rate compared with the one injection group.

Our study found no significant differences in pregnancy outcomes among four protocols for endometriosis patients. Previous research suggested that GnRHa may improve pregnancy outcomes by inhibiting hypothalamic pituitary axis function, enhancing pelvic microenvironment, and improving endometrial tolerance [5, 6]. However, another study found no significant differences in pregnancy outcomes among NC, HRT, and GnRHa-HRT protocols for FET in endometriosis patients, which was consistent with our results. Further research is needed to assess the necessity and benefits of the GnRHa-HRT protocol in FET for endometriosis patients, considering the heavier economic burden and longer duration of treatment. Similarly, our study found no significant differences in pregnancy outcomes among different protocols for endometriosis patients complicated with adenomyosis, but previous research had conflicting results [16, 17]. The small number of cases in our study and the fact that other studies primarily focused on patients with adenomyosis alone might explain the inconsistency in our findings.

Our study found that multiple injections of GnRHa in the GnRHa-HRT protocol did not improve pregnancy outcomes and might increase the risk of early miscarriage. While previous studies have been inconsistent about the use of the GnRHa ultralong protocol for IVF/ICSI in patients with endometriosis [18, 19]. The latest ESHRE guidelines did not recommend the use of ultralong protocol to improve live birth rates [4]. Other studies have also found no improvement in pregnancy outcomes with the ultralong protocol in endometriosis patients and have shown that shortened GnRHa application for IVF is equally effective [20, 21]. The reason for the increased early miscarriage rate in the two injections of GnRHa group needs further investigation. Long-term use of GnRHa can negatively affect oocyte and embryo quality [25, 26], while inadequate progesterone levels may lead to early pregnancy loss or placental defects [27, 28]. However, the cases using multiple GnRHa injections in our study was significantly less than those using one injection, so the results might be biased.

The advantages of this study were that it included the largest number of cycles compared with previous studies, and it compared all commonly used endometrial preparation protocols. For the first time, we compared pregnancy outcomes in patients with endometriosis undergoing FET with different numbers of GnRHa injections. This study was limited by its retrospective nature. The dosage and injections of GnRHa in the GnRHa-HRT protocol were not uniform, so there might be biases in interpreting the results.

## Conclusions

For infertile women with endometriosis, the clinical outcomes of different endometrial preparation protocols during the FET cycle were similar. Whether it was a single injection of GnRHa or multiple injections, full or reduced dosage of GnRHa downregulation did not improve the clinical outcomes of the FET cycle. On the contrary, multiple injections of GnRHa increased the rate of early pregnancy miscarriage. Therefore, for infertile women with endometriosis, the endometrial preparation protocol during the FET cycle might not require the GnRHa downregulation, it needs some well-designed prospective studies to further confirm.

## Data Availability

The datasets generated and/or analysed during the current study are not publicly available.

## Abbreviations

FET: Frozen-thawed embryo transfer
GnRHa-HRT: Gonadotropinreleasing hormone agonist-hormone replacement therapy
HRT: Hormone replacement therapy
OI: Ovulation induction
NC: Natural cycle
PSM: Propensity score matching
ESHRE: European Society of Human Reproduction and Embryology
ART: Assisted reproductive technology
E2: Oestradiol
P: Progesterone
LH: Luteinizing hormone
HCG: Human chorionic gonadotropin
HMG: Human menopausal gonadotropin
ASRM: American Society for Reproductive Medicine
Gn: Gonadotropin
PGT: Preimplantation genetic testing
AORs: Adjusted odds ratios

## References

[1] Coccia ME, Nardone L, Rizzello F. *Endometriosis and infertility: a Long-Life Approach to Preserve Reproductive Integrity*. Int J Environ Res Public Health, 2022. 19(10). 10.3390/ijerph19106162 PMC9141878 35627698

[2] Practice Committee of the American Society for Reproductive (2012). Endometriosis and infertility: a committee opinion. Fertil Steril.

[3] Dunselman GA (2014). ESHRE guideline: management of women with endometriosis. Hum Reprod.

[4] members of the Endometriosis Guideline Core (2022). ESHRE guideline: endometriosis. Hum Reprod Open.

[5] Beloosesky R (2000). Ovarian stimulation in in vitro fertilization with or without the long gonadotropin-releasing hormone agonist protocol: effect on cycle duration and outcome. Fertil Steril.

[6] Ferrero S (2009). GnRH analogue remarkably down-regulates inflammatory proteins in peritoneal fluid proteome of women with endometriosis. J Reprod Med.

[7] Luo L (2021). Pregnancy outcome and cost-effectiveness comparisons of artificial cycle-prepared frozen embryo transfer with or without GnRH agonist pretreatment for polycystic ovary syndrome: a randomised controlled trial. BJOG.

[8] Schmidt CL (1989). Transfer of cryopreserved-thawed embryos: the natural cycle versus controlled preparation of the endometrium with gonadotropin-releasing hormone agonist and exogenous estradiol and progesterone (GEEP). Fertil Steril.

[9] Simon A (1998). Transfer of frozen-thawed embryos in artificially prepared cycles with and without prior gonadotrophin-releasing hormone agonist suppression: a prospective randomized study. Hum Reprod.

[10] Keltz MD (1995). Baseline cyst formation after luteal phase gonadotropin-releasing hormone agonist administration is linked to poor in vitro fertilization outcome. Fertil Steril.

[11] Xu J (2021). Endometrial Preparation for frozen-thawed embryo transfer with or without pretreatment with GnRH agonist: a randomized controlled trial at two Centers. Front Endocrinol (Lausanne).

[12] Cao X (2020). The effectiveness of different down-regulating protocols on in vitro fertilization-embryo transfer in endometriosis: a meta-analysis. Reprod Biol Endocrinol.

[13] Guo Y (2023). Which endometrial preparation protocol provides better pregnancy and perinatal outcomes for endometriosis patients in frozen-thawed embryo transfer cycles? A retrospective study on 1413 patients. J Ovarian Res.

[14] Eisenberg VH et al. *Sonographic Signs of Adenomyosis Are Prevalent in Women Undergoing Surgery for Endometriosis and May Suggest a Higher Risk of Infertility* Biomed Res Int, 2017. 2017: p. 8967803. 10.1155/2017/8967803 PMC5624198 29098162

[15] Chen M (2020). Impact of Gonadotropin-Releasing hormone agonist pre-treatment on the cumulative live birth rate in Infertile Women with adenomyosis treated with IVF/ICSI: a retrospective cohort study. Front Endocrinol (Lausanne).

[16] Zhang W (2022). Effect of GnRH-a pretreatment before frozen-thawed embryo transfer on pregnancy outcome of adenomyosis-associated infertile patients with 56 cm(3) = uterine volume </=100 cm(3)</at. Ann Transl Med.

[17] Li M (2021). Effects of artificial cycles with and without gonadotropin-releasing hormone agonist pretreatment on frozen embryo transfer outcomes in patients with adenomyosis. Sci Rep.

[18] Niu Z (2013). Long-term pituitary downregulation before frozen embryo transfer could improve pregnancy outcomes in women with adenomyosis. Gynecol Endocrinol.

[19] Georgiou EX et al. *Long-term GnRH agonist therapy before in vitro fertilisation (IVF) for improving fertility outcomes in women with endometriosis*. Cochrane Database Syst Rev, 2019. 2019(11). 10.1002/14651858.CD013240.pub2 PMC6867786 31747470

[20] Tomassetti C (2021). The ultra-long study: a randomized controlled trial evaluating long-term GnRH downregulation prior to ART in women with endometriosis. Hum Reprod.

[21] Kong H (2019). Efficacy and safety of in Vitro fertilization (IVF)/Intracytoplasmic sperm injection (ICSI) among patients with Endometriosis after a shortened protocol of long-term pituitary downregulation. Med Sci Monit.

[22] Damario MA, Rock JA (1997). Classification of endometriosis. Semin Reprod Endocrinol.

[23] Li X, et al. Effects of gonadotropin-releasing hormone agonist pretreatment on frozen embryo transfer outcomes in artificial cycles: a meta-analysis. Arch Gynecol Obstet; 2022. 10.1007/s00404-022-06823-7 36266549

[24] Chang Y (2022). Association of embryo transfer type with infertility in endometriosis: a systematic review and meta-analysis. J Assist Reprod Genet.

[25] Surrey ES (2017). GnRH agonist administration prior to embryo transfer in freeze-all cycles of patients with endometriosis or aberrant endometrial integrin expression. Reprod Biomed Online.

[26] Tesarik J, Mendoza C (2002). Effects of exogenous LH administration during ovarian stimulation of pituitary down-regulated young oocyte donors on oocyte yield and developmental competence. Hum Reprod.

[27] Li C (2021). Perinatal outcomes of neonates born from different endometrial preparation protocols after frozen embryo transfer: a retrospective cohort study. BMC Pregnancy Childbirth.

[28] Vinsonneau L (2022). Impact of endometrial preparation on early pregnancy loss and live birth rate after frozen embryo transfer: a large multicenter cohort study (14 421 frozen cycles). Hum Reprod Open.

